# Cross- Wellcome Africa Asian Programmes (AAPs) Acceleration of Genomics for Escalating infectious Diseases, (CAGED) Consortium

**DOI:** 10.12688/wellcomeopenres.25620.1

**Published:** 2026-02-03

**Authors:** Patience Kerubo Kiyuka, Elizabeth M. Batty, Audrey Dubot-Pérès, Duy Pham Thanh, Anne Amulele, Patrick Mushicha, Jennifer Cornick, Ndivhuho Makhado, Alex Sigal, Anuraj Shankar, Tan Le Van, Lynette Isabella Ochola-Oyier, CAGED Consortium

**Affiliations:** 1 KEMRI-Wellcome Trust Research Programme, Kilifi, Kenya; 2 KEMRI Centre for Geographic Medicine Research Coast, Kilifi, Kilifi County, Kenya; 3 Faculty of Tropical Medicine, Mahidol University, Mahidol Oxford Tropical Medicine Research Unit, Bangkok, Bangkok, Thailand; 4 Centre for Tropical Medicine and Global Health, University of Oxford Nuffield Department of Medicine, Oxford, England, UK; 5 Unite des Virus Emergents, Universita di Corsica, Inserm, France; 6 Oxford University Clinical Research Unit, Chi Minh City, Vietnam; 7 Malawi Liverpool Wellcome Programme, Blantyr, Malawi; 8 Africa Health Research Institute, Durban, KwaZulu-Natal, South Africa

**Keywords:** chikungunya virus, dengue virus, Mycobacterium tuberculosis (Mtb), multidrug-resistant Klebsiella pneumoniae, genomic surveillance

## Abstract

The Cross-Wellcome Africa Asian Programmes (AAPs) Acceleration of Genomics for Escalating Infectious Diseases (CAGED) Consortium is a collaboration among six institutions: the Africa Health Research Institute, the Center for Infectious Disease Research in Africa, the KEMRI-Wellcome Trust Research Programme, the Malawi Liverpool Wellcome Programme, the Mahidol Oxford Tropical Medicine Research Unit and the Oxford University Clinical Research Unit. The consortium focuses on studying drug-resistant pathogens driven by climate change and posing major public health threats in Africa and Southeast Asia including chikungunya (CHIKV), dengue (DENV), multidrug-resistant *Klebsiella pneumoniae* (Kpn), and drug-resistant *Mycobacterium tuberculosis* (Mtb). In this paper, we outline the consortium goals, planned activities, discuss challenges and future directions. By leveraging cross-continent expertise, CAGED aims to unravel the molecular epidemiology of escalating infectious diseases of public health importance in Africa and Southeast Asia, and build sustainable local sequencing capacity, helping the region better prepare for future emerging infectious disease outbreaks.

## Plain Language Summary

The CAGED consortium seeks to leverage the capability of six institutions spread across Africa and South East Asia: the Africa Health Research Institute (AHRI) in South Africa, the Center for Infectious Disease Research in Africa (CIDRI-Africa) in the University of Cape Town in South Africa, KEMRI-Wellcome Trust Research Programme (KWTRP) in Kenya; the Malawi Liverpool Wellcome Programme (MLW) in Malawi; the Mahidol Oxford Tropical Medicine Research Unit (MORU) in Thailand and Laos; the Oxford University Clinical Research Unit (OUCRU) in Vietnam and Indonesia. The consortium seeks to scale up genomic surveillance for diseases driven by climate change and of public health importance in these regions. Specifically, we aim to advance multi-pathogen genomic epidemiology for characterizing transmission dynamics, tracking the emergence and spread of outbreak-associated variants, and assessing their potential to evade treatments, host immunity, and vaccines.

We will use a multi-pathogen genomic surveillance approach to study the following pathogens: chikungunya (CHIKV), dengue (DENV), multidrug-resistant *Klebsiella pneumoniae* (Kpn), and drug-resistant *Mycobacterium tuberculosis* (Mtb) to bolster pathogen sequences in this region and inform improved diagnostics for these pathogens, while delineating drug resistance markers. We will share the data analysis tools developed from this study with the National Public Health Laboratory (NPHL) to improve their genomic surveillance capabilities. Additionally, we propose working closely with the policymakers to ensure that the results generated feed into policy-making decisions.

## Introduction

Genomic surveillance has become a top priority for the World Health Organization, through their strategy to “focus on the specialized role of genomics as a cross-cutting capacity within the broader health system from a public health lens”. Genomics can provide insights into pathogen evolution, transmission, drug resistance, the emergence of clinically important variants and disease epidemiology. Such insights are critical for the development of intervention strategies and health policies as demonstrated during the COVID-19 pandemic, and equally relevant to endemic and escalating infections. Given the heterogeneity in infectious disease epidemiology in Africa and Asia, a multi-site and multi-pathogen approach across different geographical regions will provide a rich source of clinical and epidemiological data linked to genomics to enhance public health impact.

The Wellcome Africa Asian Programmes (AAPs) (know termed the Major International Programmes, MIPs) include: the Africa Health Research Institute (AHRI) in South Africa, the Center for Infectious Disease Research in Africa (CIDRI-Africa) in the University of Cape Town in South Africa, KEMRI-Wellcome Trust Research Programme (KWTRP) in Kenya; the Malawi Liverpool Wellcome Programme (MLW) in Malawi; the Mahidol Oxford Tropical Medicine Research Unit (MORU) in Thailand and Laos; the Oxford University Clinical Research Unit (OUCRU) in Vietnam and Indonesia. Together, the AAPs/CIDRI-A have a wealth of experience conducting genomic surveillance on multiple pathogens, through a single researcher led focus, such as CHIKV (KWTRP) ^1^, DENV (MORU, OUCRU) ^2, 3^, HIV (AHRI), respiratory syncytial virus (KWTRP) ^4^, Kpn (MLW, MORU, OUCRU) ^5– 7^, malaria (KWTRP, MORU, OUCRU) ^8^, metagenomics (OUCRU) ^9^ and Mtb (AHRI, CIDRI-A, OUCRU) ^10, 11^. The sequencing of these pathogens at all the sites is primarily conducted in-house on multiple next-generation sequencing platforms, such as Illumina and Oxford Nanopore Technology (ONT), anchored on strong platforms of infectious disease epidemiology, detailed surveillance to interrogate the impact of these infections in cohorts, communities and health facilities, while also examining the biology and immunology of the infections to inform drug and vaccine design. Furthermore, our rich resource of archived samples linked to well-characterized clinical and epidemiological data informs the historical context of these infectious diseases. Notably, we have conducted amplicon-based, whole genome and agnostic metagenomics sequencing, allowing us to overcome technical challenges posed by the diversity of the pathogens. This is supported by established bespoke pathogen specific bioinformatics workflows. Thus, we can move rapidly from samples to data analysis and policy impact.

Collectively we used SARS-CoV2 genomic surveillance to determine national and subnational routes of entry and dispersion ^12, 13^ and the drivers of regional transmission, while regularly sharing the data with public health professionals and policy makers. These are the similar analyses conducted on the single-focus pathogens to define, for example, the emergence and trends of drug resistance markers locally and regionally, and the outbreak and transmission of new variants ^14^. We are proposing to scale up and deploy this expertise to a multi-pathogen genomic surveillance approach to bolster our research capacity in the four pathogens, while maximizing on our strategic geographic locations to ensure research uptake into policy.

We selected high burden endemic diseases CHIKV, DENV, Kpn and Mtb, with a currently limited use of genomics to support disease surveillance efforts, in Africa and Asia. This is in comparison to over 3 million SARS-CoV-2 genomes deposited on GISAID since the beginning of the COVID-19 pandemic ^15^. DENV is ubiquitous in the tropics, causing around 390 million infections globally every year, however only 96 million are symptomatic ^16^. Currently, there are 3.9 billion people at risk of contracting DENV, 70% of whom are from the Western Pacific and South-East Asia regions ^17^. In Africa, the estimated number of DENV infections exceeds 60 million ^18^, and half a million people require hospitalization as per WHO estimates, with a ~2.5% mortality rate ^19^.

CHIKV which initially caused small outbreaks, has recently expanded beyond the Indian Ocean Islands ^20^. Since, its initial description in Tanzania in 1952 ^21^ one of the largest epidemics, in 2004, resulted from its re-emergence in Coastal Kenya affecting millions of people and spreading along the Indian Ocean islands, India, Southeast Asia and Europe, with a new emergent lineage ^22, 23^. CHIKV mortality rates are low (~0.1%), however morbidity has a substantial impact on the quality of life as the virus can lead to neurological disease and chronic disability ^24, 25^. CHIKV and DENV symptoms are similar, and the infections are often confused ^26^, thus molecular diagnostics are an imperative.

Hospital and community acquired Kpn infections in Asia and Africa, predominantly affect neonates, young children and adults in intensive care units (ICUs) ^27^. The burden of disease is exacerbated by the emergence and spread of multidrug-resistant (MDR) and hypervirulent Kpn strains ^28^. Kpn is the second leading cause of death due to antimicrobial resistance (AMR), with over 200,000 fatalities attributed to AMR in 2019 ^29^. The accumulation of AMR is primarily due to horizontal gene transfer aided by plasmids and mobile genetic elements ^28, 30^. These resistance mechanisms can be interrogated using whole genome data. For Mtb, the treatment success for rifampicin-resistant TB (RR-TB) is ~63%, however of the ~410,000 patients with RR-TB in 2022, only 2 in 5 people were enrolled on RR-TB therapy ^31^. In 2022, the WHO announced that RR-TB could be treated with a new regimen of bedaquiline (B), pretomanid (Pa), linezolid (L) (BPaL), with or without moxifloxacin (M) ^32^. The BPaL(M) regimen is up to 50% more effective than the previous standard-of-care, it is fully oral and given over a shorter (6 months) time period ^33^. This regimen is prescribed in at least 40 countries, but without an adequate companion molecular diagnostic to monitor resistance ^31^. Though in 2023, targeted next-generation sequencing was recommended by WHO for RR-TB ^34^.

The MIPs will collectively utilise their broad expertise, lessons from the COVID-19 pandemic and their current research strengths, to provide comprehensive genetic profiles for these four pathogens. More importantly, we will work towards integrating the genomic surveillance by moving platforms developed and data generated to the National Public Health Laboratory (NPHL) such that the data is used to accelerate evidence-based policy decision making, for instance, antimicrobial stewardship. The aim of the project is to advance multi-pathogen genomic epidemiology for characterizing transmission dynamics, tracking the emergence and spread of outbreak-associated variants and assessing their potential to evade treatments, host immunity and vaccines. This will be done through; coordinating and synergizing the strengths across AAPs/CIDRI-A and data integration of all 4 pathogens; determining the transmission networks of chikungunya and dengue outbreaks and the potential of these rapidly evolving viruses to escape host immunity and vaccination; determining the emergence and transmission of multidrug-resistant *Klebsiella pneumoniae* in hospital facilities and community settings; and determining the emergence of resistance to bedaquiline, pretomanid, linezolid and moxifloxacin among rifampicin resistant Mtb strains.

## Organization of the consortium

### The consortium is organized into 5 work packages

***Work package 1: Project management***

The consortium operates under a transparent governance framework to ensure effective coordination and management of the programs of work across participating institutions. Overall leadership is shared between two coordinating institutions KWTRP and OUCRU.

**Figure 1.**
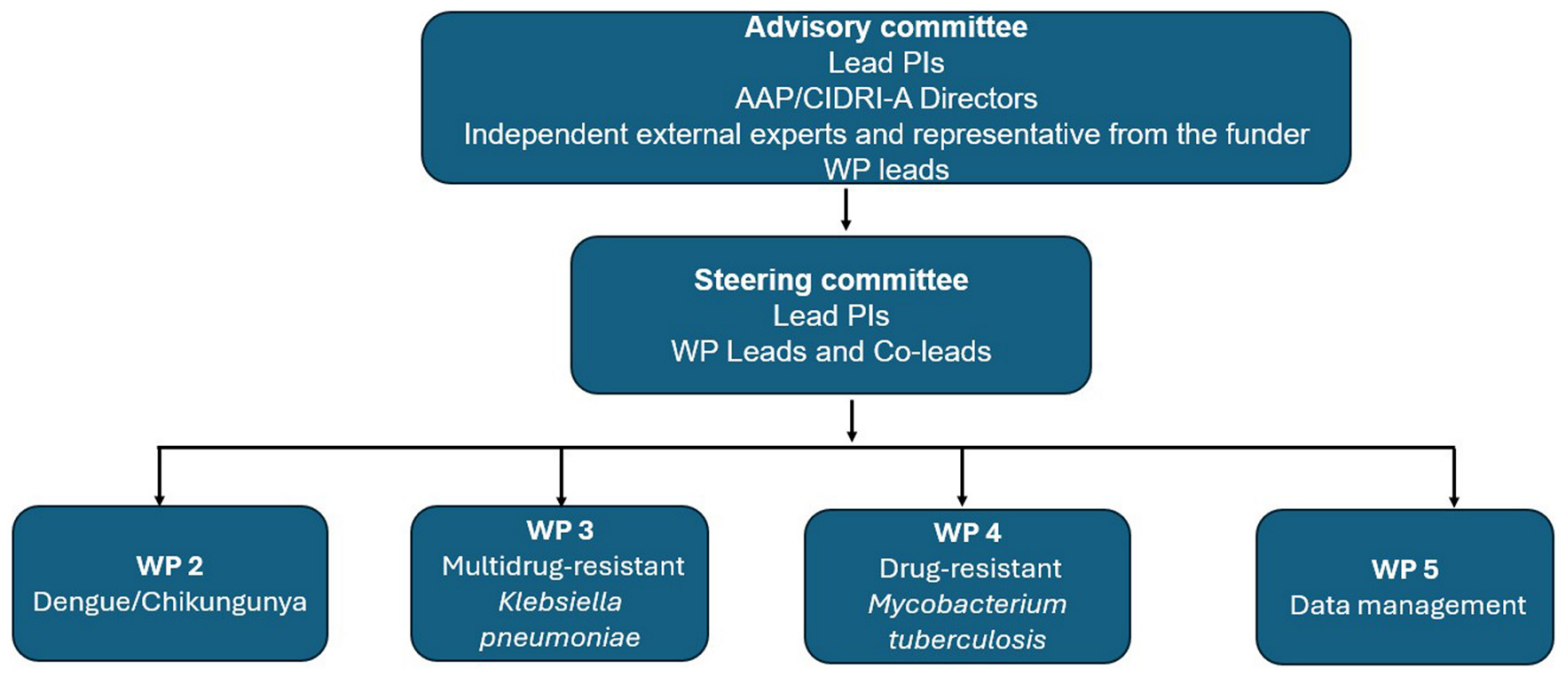
Figure 1 shows the project organogram.

The consortium's governance is organized across three primary committees to ensure strategic oversight, operational management, and support for execution of the project activities Figure 1. The Advisory Committee (AC) serves as the highest decision-making committee. The AC is composed of the Directors of the AAP/CIDRI-A, a selection of independent external experts, and an observer from the Wellcome Infectious Disease Strategic team. The AC serves to provide strategic direction, scientific critique and policy guidance on consortium activities.

The project will be managed by the Steering Committee (SC), who are the lead applicants, co-applicants and WP leads from the AAPs/CIDRI-A. The SC's will: oversee and set the priorities and scope of research activities of the consortium, manage project resources, and coordinate the various Work Packages. The SC will convene for quarterly virtual meetings and once a year. Its responsibilities also extend to actively engaging with policymakers and producing quarterly reports on project outputs for dissemination to stakeholders.

The consortium research activities are carried out through Work Packages (WPs). Each WP is led by a designated Lead and Co-Lead, and relevant members from each AAP/CIDRI-A. These teams are responsible for developing and implementing detailed work plans, engaging stakeholders and collaborators to implement project activities, reviewing whole-genome sequencing (WGS) assays and bioinformatics pipelines, conducting country-level and cross AAPs/CIDRI-A data analysis, and holding monthly coordination meetings to ensure harmonised progress.

Quarterly reports will be generated to evaluate project progress at all sites. It will include information on samples, sources and sequencing, variants detected, expenditure, dissemination activities, publications, health facility reports, and policy briefs shared and stakeholder engagements.

## Methodology

The pathogen specific work packages will be delivered through a harmonised analytical approach linked through sharing of the resources. We will build on existing investments in sample collection and surveillance to analyse archived and prospective samples that we have established through long-term local partnerships Table 2. Thus, ethical consideration has already been obtained for these studies for future work or for molecular analyses.

All archived samples will form part of the retrospective observational study for each pathogen and will be selected based on the availability of requisite meta-data to support the genomic epidemiology analyses. All prospective samples from ongoing studies in these sites will be obtained from consenting participants. A standard data collection tool will be used across all AAPs/CIDRI-A for each pathogen to support easy access, data sharing and analyses. Table 1 lists details of the samples to be used for this study.

**Table 1.** Description of the samples to be used for this study.

Work package	Site	Source of sample and sample type	Sample type
Chikungunya and Dengue	MORU	Hospital samples	Blood
	KWTRP	Archived blood samples in the biobank at KWTRP and from collaborators such as NPHI and KEMRI CVR	Blood
	AHRI	National Health Laboratory Service of South Africa and AHRI-Durban cohort of participants with respiratory and febrile illness based at Inkosi Albert Luthuli Central Hospital, King Edward Hospital, Clairwood Hospital, and KwaDabeka community clinic	Blood
	MLW	Multiple health facilities and from ongoing studies under the vector programme	Mainly serum and plasma as well as NP and CSF, mosquitoes
*Klebsiella* *pneumoniae* (Kpn)	COMRU	Angkor Hospital for Children	Blood
	KWTRP	Kilifi County Hospital (neonates and paediatrics (retrospective and prospective, from HDU and normal wards) adults (retrospective from wards)), Mbagathi County Hospital, Nairobi (neonates and paediatrics from neonate and children wards and the acute room (not exactly a HDU)), Community health facilities in Kilifi (3 health centres catering to the community) (children and adults)	Blood, urine, CSF
	CIDRI-A	Tertiary referral hospital for Infectious diseases, tertiary general hospital and community carriage studies	
	MLW	Queen Elizabeth Central Hospital, Zomba Central Hospital	Blood, CSF
	OUCRU	Tertiary referral hospital for Infectious diseases & tertiary general hospital	Blood, Urine
*Mycobacterium* * tuberculosis* (Mtb)	SMRU	Hospitals and centralised DR-TB clinics	Sputum and cultures
	KWTRP	Kilifi County Hospital, Malindi sub-county Hospital and Mariakani sub-county hospital	
	AHRI	National Health Laboratory Service of South Africa and AHRI-Somkhele Demographic Surveillance Site based in uMkhanyakude district, KwaZulu-Natal province	Sputum and isolated cultures
	CIDRI-A	Nkqubela Chest Hospital, a public referral hospital in the Buffalo City Municipality, East London, Eastern Cape, South Africa	

Mahidol Oxford Tropical Medicine Research Unit (MORU); KEMRI-Wellcome Trust Research Programme (KWTRP); Africa Health Research Institute (AHRI); Malawi (MLW); Center for Infectious Diseases Research in Africa (CIDRI-A); Oxford University Clinical Research Unit (OUCRU); Cambodia-Oxford Medical Research Unit (COMRU); Shoklo Malaria Research Unit (SMRU)

**Table 2.** Project timelines and milestones.

Activity	Year 1		Year 2		Year 3	
	Jan-Jun	Jul-Dec	Jan-Jun	Jul-Dec	Jan-Jun	Jul-Dec
**Work package 2, CHIKV & DENV**						
*Sub-Aim 2.1 generation of a centralized sample database*						
Setting up central database						
Harmonization of sequencing analysis plan						
Laboratory sample analysis using RT-PCR						
*Sub-Aim 2.2 capacity development and sequencing*						
Setting up CHIKV & DENV sequencing at all sites						
Sample RNA extraction and whole genome sequencing (WGS) using Oxford Nanopore or Illumina						
*Sub-Aim 2.3 sequence analysis and tracking variants*						
Sequence alignment generation						
Phylogenetic analysis to define lineages and new variants						
Overall cross AAPs/CIDRI-A sequence analysis and submission to GenBank and GISAID						
*Sub-Aim 2.4 characterization of neutralising antibody responses*						
Setting up neutralisation assays across the AAPs						
Overall serological analysis (antigenic cartography)						
**Work package 3, *Klebsiella pneumoniae***						
*Sub-Aim 3.1 generation of a centralized sample database*						
Setting up central database						
Prospective sample collection, blood and CSF						
Harmonization of Kpn Illumina and OTN sequencing and bioinformatics pipelines						
*Sub-Aim 3.2 capacity development and sequencing*						
Setting up Kpn sequencing at all sites						
Sample DNA extraction and WGS						
*Sub-Aim 3.3 sequence analysis and tracking variants*						
Sequence alignment generation						
Phylogenetic analysis to define lineages and new variants						
Overall cross AAPs/CIDRI-A sequence analysis and submission to Kleborate and Pathogenwatch						
*Sub-Aim 3.4 Characterisation of antimicrobial resistance (AMR)*						
Identification of clinically relevant MDR lineages in Africa and Sout East Asia						
Detection of plasmids, AMR genes and hypervirulent lineages						
Support response to local outbreaks and check for association between hospital and community Kpn carriage						
Data sharing via Pathogenwatch						
Dissemination of results and stakeholder engagement						
**Work package 4, *Mycobacterium tuberculosis (Mtb)***						
*Sub-Aim 4.1 Generation of a centralized sample database*						
Setting up central database						
Harmonize sequence analysis plan						
Prospective sample collection and Xpert RR-TB screening						
Harmonize protocols for Mtb Illumina/OTN sequencing and bioinformatics pipelines						
*Sub-Aim 4.2 Capacity development and sequencing*						
Training and setting up Mtb sequencing at all sites						
Sample DNA extraction and WGS						
*Sub-Aim 4.3 sequence analysis and tracking variants*						
Sequence alignment generation						
Phylogenetic analysis to define lineages and new variants						
Overall cross AAPs/CIDRI-A sequence analysis and submission to MTBseq and Pathogenwatch						
*Sub-Aim 4.4 Characterisation of AMR*						
Identification of clinically relevant MDR lineages						
Identification of Mtb lineage differences between Africa and SE Asia						
Determination of local emergence and prevalence of drug resistance mutations						
Data sharing via Pathogenwatch						
Dissemination of results and stakeholder engagement						
**Work package 5, Data management**						
Agreement of data collection variables						
Training on FHIR standard						
Integration of current AAPs/CIDRI-A data platforms into the FHIR standard						
Sequence data submitted to GenBank, Nextstrain, EMBL						
Bioinformatics pipelines made available on GitHub						

### Chikungunya (CHIKV) and Dengue (DENV) work package

We will utilise retrospective and prospective confirmed (by Real-Time (RT)-quantitative PCR CHIKV blood samples. Additional febrile surveillance retrospective and prospective samples will be screened by RT-qPCR for CHIKV and DENV. Sequencing and bioinformatics pipeline methodologies will be standardized across the sites. WGS of CHIKV and DENV will be done on the Illumina and ONT platforms for CHIKV using a pooled amplicon approach and DENV established Illumina methods.

Consensus genomes will be generated and both maximum likelihood and Bayesian phylogenetic trees will then be constructed in the context of global CHIKV and DENV sequences obtained from GenBank, representing the 3 distinct CHIKV clades from East/Central/South Africa, West Africa and Asia. For DENV, the representative sequences will include the four distinct serotype strains DENV-1, 2, 3 and 4. We aim to identify distinct CHIKV and DENV lineages, global transmission trends and detect any new emerging outbreak strains.

To assess neutralizing antibodies against CHIKV and DENV a minimum of 20 participant samples per group will be selected to examine strain differences related to immune/vaccine escape. Representative live virus strains from each location, as informed by phylogenetic analysis, will be isolated and tested against geography specific sera from convalescent individuals (~3 months following the initial febrile episode). We will perform multiplexed serological epitope mapping approaches using data from live virus neutralization assays, to discern correlates of immune protection and escape across strains ^35, 36^. The geometric mean titres per group of sera against each DENV and CHIKV viral isolate will be determined and the Racmacs package for antigenic cartography will be used to determine antigenic distance between viral isolates ^37, 38^.

***Expected outcomes***

The WGS data will be deposited on GenBank (doubling the number of genomes) and GISAID. The phylogenetic analysis of historical and contemporary CHIKV and DENV sequences will identify new lineages, the spread of lineages across Asia and Africa and potential imports within each geographic location. The neutralisation assays will provide a better understanding of the potential protective effect of DENV and CHIKV vaccines given the DENV and CHIKV genetic diversity defined in this project and its consequences for immune escape.

***Klebsiella pneumoniae* (Kpn) work package**

We will randomly select Kpn positive samples collected from hospital and community carriage surveillance. We will utilize an equivalent number of Kpn community carriage strains as hospital facilities (HF) strains. We will also leverage existing surveillance efforts such as the Wellcome-funded A Clinically Oriented Antimicrobial Resistance Network (ACORN) study ^39^ to collect patient, microbiology and clinical metadata using the available standard protocols to ensure data harmonization. In addition, we will collect information on any previous and current antibiotics prescribed.

We will use a single whole genome sequencing (WGS) standard operating procedure (SOP) and bioinformatics pipeline. Following Kpn identification by blood and CSF culture and the routine antimicrobial susceptibility testing to support clinical care, all positive Kpn samples will be cultured to provide sufficient DNA for extraction. WGS will be done on the Illumina MiSeq and the OTN platform for selected samples.

We will use well-established bioinformatics tools such as Kleborate ^40^ for the characterisation of Kpn genomes including multilocus sequence typing (MLST), delineating the Kpn complex into species, screening for AMR and virulence genes and the mobile elements (including plasmids) harbouring them. We will use Kleborate with Kaptive ^41^ to identify capsule K and the lipopolysaccharide O antigen loci. Short read sequence typing (SRST2) for bacterial pathogens ^42^, will determine the emergence of MDR and/or hypervirulent strains/variants. The genomic and clinical metadata will be uploaded to Pathogenwatch ( https://pathogen.watch), a comprehensive global platform dedicated to genomic surveillance of microbial pathogens, including 33,000 Kpn publicly available genomes, and featuring real-time analytics. Genomic data will be analyzed within the local, regional, and global contexts, including clinical parameters, enabling visualization and real-time data sharing across the sites.

We will infer transmission using phylodynamic modelling and SNP analyses. The phylogenetic analyses will identify clinically relevant lineages that emerge within a HF, network of HFs and the community that could cause a local outbreak. The data across-sites will determine whether a MDR clonal outbreak in SE Asia seeds MDR outbreaks in Africa and vice versa. We will also define the prevalence of sequence types, serotypes, AMR genes and virulence factors circulating in the different countries, hospitals and the community. These Kpn components will then be associated with clinical outcomes and the antimicrobial sensitivity test data, to determine which components are associated with, for instance, third-generation cephalosporin-resistance to inform treatment strategies in our populations. Similarly, the prevalence of hypervirulent clones will be defined in SE Asia and particularly in Africa where this data is limited.

***Expected outcomes***

WGS data will be on the ACORN platform and Pathogenwatch for easy data sharing with collaborators and the Ministry of Health (MoH). The archived samples will provide insights into the temporal emergence of variants. The in-depth phylogenetic analyses will elucidate the links between community carriage and hospital transmission, national and international transmission patterns of MDR strains between Asia and Africa and characterize potential outbreaks. Furthermore, we will describe the genetic elements (genes, plasmids, chromosomal insertions) associated with AMR and virulence and determine the transmission of these elements within and between strains/variants, both at a national and international scale.

***Mycobacterium tuberculosis* (Mtb) work package**

We will focus on prospective sampling from Mtb clinics within level 3 HFs and above. The standard data variables will be agreed upon across institutions and data on previous Mtb episodes and Mtb treatments and use of BPaL(M) drugs will be collected. Sputum samples will be cultured in category 3 laboratory facilities at the AAPs/CIDRI-A to obtain and extract Mtb DNA. We will use the Illumina and ONT platforms and the bioinformatics analyses will be based on a standardised and validated pipelines familiar to CIDRI-A and OUCRU, MTBseq.

Consensus genomes will be generated and variants called using an automated pipeline, MTBseq ^42^. For historical and wider geographic context, we will select additional publicly available genomes for comparison. This will identify resistance mutations relevant to fluoroquinolones, bedaquiline, pretomanid and linezolid using the WHO catalogue of mutations associated with drug resistance in Mtb ^44^. Lineages associated resistance mutations will be determined using an automated pipeline. Similar to Kpn, we will use Pathogenwatch for the cross-site analysis since it supports easy data sharing.

Phenotypic drug susceptibility testing will be performed for isolates/drugs with mutations of uncertain significance in key genes, as determined by the WHO catalogue ^43^. In addition, a logistic regression analysis will be conducted to assess epidemiological risk factors for the presence of drug resistance mutations. Time-scaled, Bayesian phylogenetic reconstruction will also be used to estimate the date of emergence of drug resistance mutations. This will form the basis for the discovery, identification and prevalence of drug resistance variants to bedaquiline, pretomanid, linezolid, and moxifloxacin.

***Expected outcomes***

The data will be on Pathogen watch for easy data sharing with our collaborators and the MoH. The in-depth phylogenetic analyses will elucidate the national and international transmission patterns of MDR strains between Asia and Africa and characterize potential outbreaks. We will describe the mutations associated with MDR-Mtb and their prevalence. Expertise in the use of MTBseq will be developed as an additional tool for the bioinformatics analyses of WGS data from clinical Mtb strains. This WP will advance resistance prediction, provide genomic MDR-Mtb genomic surveillance data to support the diagnosis of MDR-Mtb infections (those not identified by Xpert MTB/RIF) and minimise treatment failures. Thus, it will improve the optimal treatment of patients and reduce MDR-TB resistance transmission.

### Data management work package

*Through this project we aim to ensure FAIR data principles*

*Findable:* we will aim to employ standardised metadata policies and formats for example those developed by the PHA4GE network. The sequence data will be deposited on additional publicly available repositories such as Nextstrain, European Nucleotide Archive and GenBank. The standardized bioinformatic pipelines will be made available on Github.

*Accessible:* we will utilize both paper and digital systems, predominantly REDCap, Open data kit, Kobo Toolbox, and CommCare, and a mix of on-premise and cloud-based servers. We will use openly accessible data formats, with licensing that supports broader usage while respecting personal data privacy.

*Interoperable:* To harmonize these systems and link the data with the national TB, AMR and vector borne diseases programs dashboards or health information services, we will utilize the HL7 Fast Healthcare Interoperability Resources (FHIR) ^44^ data standard that is endorsed by WHO to enable data interoperability. The MoH of each country has initiated transition to the FHIR standard, with most having drafted FHIR implementation guides (IG). We will adopt and expand these to integrate our genomics and clinical meta-data. Each site will map their current and planned data schemas to their country-level FHIR IG, and we will jointly agree on harmonization encoding across countries. This process will be facilitated by engagement with the FHIR HL7 genomics working group and the FHIR GenomeX initiative, and with specific tools, such as REDCap’s Clinical Data Interoperability Services (CDIS). We will deploy the FHIR HL7 clinical quality language (CQL) or real time assessment of data quality and completeness.

*Reusable:* Documentation (data dictionaries, user manuals) will accompany each dataset, making re-analysis or meta-analysis straightforward for external researchers. Whenever feasible, we aim for a CC-BY 4.0 license, except were restricted by ethical constraints.

***Expected outcome***

Ultimately, we hope that our project will produce interoperable data in line with the FHIR standards and establish mechanisms across the sites for a federated data analysis pipeline.

### Opportunities, challenges and future directions

The CAGED study consortium serves as an excellent platform for anchoring additional research and capacity building across its sites. This foundation has already proven instrumental in securing further grant funding, including resources to expand the consortium's work into Zika virus sequencing. Capacity building efforts leverage the platform to support short-term MSc projects and partial PhD projects.

Operating across diverse partner institutions and global regions necessitates navigating country-specific regulations, including protocol approvals, data and sample sharing mechanisms, and institutional bureaucracies related to Material Transfer Agreements (MTAs).

While challenging these processes present valuable opportunities to strengthen collaborative frameworks and enhance our standing in international research. To ensure effective coordination, the consortium has established regular communication channels and formed an advisory committee. This committee, comprising heads of all partner institutions and key external stakeholders, secures high-level institutional buy-in and strategic guidance for consortium activities.

Looking beyond the current project cycle, future directions include individual countries' National Public Health Institutes and Ministries of Health. This ongoing collaboration is crucial to ensure the evidence generated directly informs national policy decisions.

### Communication/engagement and dissemination plans

The consortium activities will be implemented across three years for all the pathogen specific activities. The overall aim is to strengthen national public health institutions by providing enhanced genomic surveillance data on priority pathogens and improving outbreak response mechanisms. To achieve this, we will engage regularly with national and subnational policymakers, fostering stronger collaboration and sharing project outcomes. Additionally, partners are committed to promptly publishing results and refining the collaboration’s dissemination strategy through quarterly steering committee meetings

### Workplan

Table 2 indicates the project timelines and milestones.

## Ethics and consent

Ethical approval and consent were not required for this study.

## Disclaimer

The views expressed in this article are those of the author(s). Publication in Wellcome Open Research does not imply endorsement by Wellcome.

## Data Availability

No data are associated with this article.

## References

[1] Nyamwaya DK Otiende M Omuoyo DO : Endemic Chikungunya fever in Kenyan children: a prospective cohort study. *BMC Infect Dis.* 2021;21(1): 186. 10.1186/s12879-021-05875-5 33602147 PMC7889702

[2] Castonguay-Vanier J Klitting R Sengvilaipaseuth O : Molecular epidemiology of Dengue Viruses in three provinces of Lao PDR 2006-2010. *PLoS Negl Trop Dis.* 2018;12(1): e0006203. 10.1371/journal.pntd.0006203 29377886 PMC5805359

[3] Dang TT Pham MH Bui HV : First full-length genome sequence of Dengue Virus serotype 2 circulating in Vietnam in 2017. *Infect Drug Resist.* 2020;13:4061–4068. 10.2147/IDR.S275645 33204123 PMC7667145

[4] Owuor DC de Laurent ZR Nyawanda BO : Genetic and potential antigenic evolution of influenza a(H1N1)pdm09 viruses circulating in Kenya during 2009– 2018 influenza seasons. *Sci Rep.* 2023;13(1): 22342. 10.1038/s41598-023-49157-3 38102198 PMC10724140

[5] Chomkatekaew C Thaipadungpanit J Hearn P : Detection of maternal transmission of resistant gram-negative bacteria in a Cambodian hospital setting. *Front Microbiol.* 2023;14: 1158056. 10.3389/fmicb.2023.1158056 37125167 PMC10140293

[6] Musicha P Msefula CL Mather AE : Genomic analysis of *Klebsiella pneumoniae* isolates from Malawi reveals acquisition of multiple ESBL determinants across diverse lineages. *J Antimicrob Chemother.* 2019;74(5):1223–1232. 10.1093/jac/dkz032 30778540 PMC6477993

[7] Trang NHT Nga TVT Campbell JI : The characterization of ESBL genes in Escherichia coli and Klebsiella pneumoniae causing nosocomial infections in Vietnam. *J Infect Dev Ctries.* 2013;7(12):922–928. 10.3855/jidc.2938 24334938

[8] Osoti V Akinyi M Wamae K : Targeted amplicon deep sequencing for monitoring antimalarial resistance markers in Western Kenya. *Antimicrob Agents Chemother.* 2022;66(4): e0194521. 10.1128/aac.01945-21 35266823 PMC9017353

[9] Tan LV Hong NTT Ngoc NM : SARS-CoV-2 and co-infections detection in nasopharyngeal throat swabs of COVID-19 patients by metagenomics. *J Infect.* 2020;81(2):e175–e177. 10.1016/j.jinf.2020.06.033 32562797 PMC7403860

[10] Srinivasan V Ha VTN Vinh DN : Sources of multidrug resistance in patients with previous isoniazid-resistant tuberculosis identified using whole genome sequencing: a longitudinal cohort study. *Clin Infect Dis.* 2020;71(10):e532–e539. 10.1093/cid/ciaa254 32166306 PMC7744982

[11] Wasserman S Louw G Ramangoaela L : Linezolid resistance in patients with drug-resistant TB and treatment failure in South Africa. *J Antimicrob Chemother.* 2019;74(8):2377–2384. 10.1093/jac/dkz206 31081017 PMC6640298

[12] Githinji G de Laurent ZR Mohammed KS : Tracking the introduction and spread of SARS-CoV-2 in coastal Kenya. *Nat Commun.* 2021;12(1): 4809. 10.1038/s41467-021-25137-x 34376689 PMC8355311

[13] Joonlasak K Batty EM Kochakarn T : Genomic surveillance of SARS-CoV-2 in Thailand reveals mixed imported populations, a local lineage expansion and a virus with truncated ORF7a. *Virus Res.* 2021;292: 198233. 10.1016/j.virusres.2020.198233 33227343 PMC7679658

[14] Chau NVV Thuong TC Hung NT : Emerging enterovirus A71 subgenogroup B5 causing severe hand, foot, and mouth disease, Vietnam, 2023. *Emerg Infect Dis.* 2023;30(2):363–367. 10.3201/eid3002.231024 38270132 PMC10826755

[15] Fumagalli SE Padhiar NH Meyer D : Analysis of 3.5 million SARS-CoV-2 sequences reveals unique mutational trends with consistent nucleotide and codon frequencies. *Virol J.* 2023;20(1): 31. 10.1186/s12985-023-01982-8 36812119 PMC9936480

[16] Brady OJ Gething PW Bhatt S : Refining the global spatial limits of Dengue Virus transmission by evidence-based consensus. *PLoS Negl Trop Dis.* 2012;6(8): e1760. 10.1371/journal.pntd.0001760 22880140 PMC3413714

[17] WHO: Dengue and severe dengue.2024; Accessed May 15, 2025. Reference Source

[18] Bhatt S Gething PW Brady OJ : The global distribution and burden of dengue. *Nature.* 2013;496(7446):504–507. 10.1038/nature12060 23563266 PMC3651993

[19] WHO A: Dengue|WHO|Regional Office for Africa. May 14,2025; Accessed May 15, 2025. Reference Source

[20] Weaver SC Forrester NL : Chikungunya: evolutionary history and recent epidemic spread. *Antiviral Res.* 2015;120:32–39. 10.1016/j.antiviral.2015.04.016 25979669

[21] Ross RW : The Newala epidemic. III. The virus: isolation, pathogenic properties and relationship to the epidemic. *J Hyg (Lond).* 1956;54(2):177–191. 10.1017/s0022172400044442 13346078 PMC2218030

[22] Chretien JP Anyamba A Bedno SA : Drought-associated Chikungunya emergence along coastal East Africa. *Am J Trop Med Hyg.* 2007;76(3):405–407. 17360859

[23] Wahid B Ali A Rafique S : Global expansion of Chikungunya Virus: mapping the 64-year history. *Int J Infect Dis.* 2017;58:69–76. 10.1016/j.ijid.2017.03.006 28288924

[24] Renault P Josseran L Pierre V : Chikungunya-related fatality rates, Mauritius, India, and Reunion Island. *Emerg Infect Dis.* 2008;14(8): 1327. 10.3201/eid1408.080201 18680676 PMC2600376

[25] Schilte C Staikovsky F Couderc T : Chikungunya Virus-associated long-term arthralgia: a 36-month prospective longitudinal study. *PLoS Negl Trop Dis.* 2013;7(3): e2137. 10.1371/journal.pntd.0002137 23556021 PMC3605278

[26] Halstead SB : Reappearance of Chikungunya, formerly called Dengue, in the Americas. *Emerg Infect Dis.* 2015;21(1):557–61. 10.3201/eid2104.141723 25816211 PMC4378492

[27] Podschun R Ullmann U : **Klebsiella** spp. As nosocomial pathogens: epidemiology, taxonomy, typing methods, and pathogenicity factors. *Clin Microbiol Rev.* 1998;11(4):589–603. 10.1128/CMR.11.4.589 9767057 PMC88898

[28] Pendleton JN Gorman SP Gilmore BF : Clinical relevance of the ESKAPE pathogens. *Expert Rev Anti Infect Ther.* 2013;11(3):297–308. 10.1586/eri.13.12 23458769

[29] Antimicrobial Resistance Collaborators: Global burden of bacterial antimicrobial resistance in 2019: a systematic analysis. *Lancet.* 2022;399(10325):629–655. 10.1016/S0140-6736(21)02724-0 35065702 PMC8841637

[30] Wyres KL Holt KE : *Klebsiella pneumoniae* as a key trafficker of drug resistance genes from environmental to clinically important bacteria. *Curr Opin Microbiol.* 2018;45:131–139. 10.1016/j.mib.2018.04.004 29723841

[31] WHO: Global tuberculosis report 2023.2023; Accessed May 15, 2025. Reference Source

[32] WHO: Rapid communication: key changes to the treatment of drug-resistant tuberculosis.2022; Accessed May 15, 2025. Reference Source

[33] Conradie F Bagdasaryan TR Borisov S : Bedaquiline–pretomanid–linezolid regimens for drug-resistant tuberculosis. *N Engl J Med.* 2022;387(9):810–823. 10.1056/NEJMoa2119430 36053506 PMC9490302

[34] WHO: Use of targeted next-generation sequencingto detect drug-resistant tuberculosis.2023; Accessed May 15, 2025. Reference Source

[35] Khan K Karim F Ganga Y : Omicron BA.4/BA.5 escape neutralizing immunity elicited by BA.1 infection. *Nat Commun.* 2022;13: 4686. 10.1038/s41467-022-32396-9 35948557 PMC9364294

[36] Khan K Lustig G Römer C : Evolution and neutralization escape of the SARS-CoV-2 BA.2.86 subvariant. *Nat Commun.* 2023;14: 8078. 10.1038/s41467-023-43703-3 38057313 PMC10700484

[37] Lustig G Ganga Y Rodel HE : SARS-CoV-2 infection in immunosuppression evolves sub-lineages which independently accumulate neutralization escape mutations. *Virus Evol.* 2023;10(1): vead075. 10.1093/ve/vead075 38361824 PMC10868398

[38] Smith DJ Lapedes AS de Jong JC : Mapping the antigenic and genetic evolution of influenza virus. *Science.* 2004;305(5682):371–376. 10.1126/science.1097211 15218094

[39] Mo Y Ding Y Cao Y : AACORN (A Clinically-Oriented Antimicrobial Resistance Surveillance Network) II: protocol for case based antimicrobial resistance surveillance [version 2; peer review: 2 approved]. *Wellcome Open Res.* 2023;8:179. 10.12688/wellcomeopenres.19210.2 37854055 PMC10579854

[40] Lam MMC Wick RR Watts SC : A genomic surveillance framework and genotyping tool for *Klebsiella pneumoniae* and its related species complex. *Nat Commun.* 2021;12(1): 4188. 10.1038/s41467-021-24448-3 34234121 PMC8263825

[41] Wick RR Heinz E Holt KE : Kaptive web: user-friendly capsule and lipopolysaccharide serotype prediction for *Klebsiella* genomes. *J Clin Microbiol.* 2018;56(6): e00197-18. 10.1128/JCM.00197-18 29618504 PMC5971559

[42] Inouye M Dashnow H Raven LA : SRST2: rapid genomic surveillance for public health and hospital microbiology labs. *Genome Med.* 2014;6(11): 90. 10.1186/s13073-014-0090-6 25422674 PMC4237778

[43] WHO: Catalogue of mutations in Mycobacterium tuberculosis complex and their association with drug resistance, 2nd ed.2023; Accessed May 15, 2025. Reference Source

[44] Vorisek CN Lehne M Klopfenstein SAI : Fast Healthcare Interoperability Resources (FHIR) for interoperability in health research: systematic review. *JMIR Med Inform.* 2022;10(7): e35724. 10.2196/35724 35852842 PMC9346559

