# Supplementary material for: Cross- Wellcome Africa Asian Programmes (AAPs) Acceleration of Genomics for Escalating infectious Diseases, (CAGED) Consortium
Source: Wellcome Open Res. 2026 Jun 5;11:87. Originally published 2026 Feb 3. [Version 2] doi: 10.12688/wellcomeopenres.25620.2 (PMC13264690; doi:10.12688/wellcomeopenres.25620.2)
Supplement: Peer review history

# Reviewer report: Reviewer response for version 2

**DOI:** 10.21956/wellcomeopenres.29469.r157700

**Published:** 2026-06-13

**Authors:** Gerald Mboowa

**Affiliations:** 1 Makerere University, Kampala, Uganda

I have reviewed the revised version of this Open Letter. The authors have addressed the major concerns raised in the previous review and have substantially improved the clarity, rationale and structure of the current manuscript.

 The revised manuscript now provides a clearer justification for the CAGED consortium, including the public health importance of the selected pathogens, the need for multi-pathogen genomic surveillance across Africa and Southeast Asia, and the importance of strengthening local sequencing and bioinformatics capacity. The added information on current genomic data availability, existing surveillance platforms, surveillance gaps, and country-level differences strengthens the rationale for the work.

 The work packages are now described more clearly, including project governance, CHIKV/DENV genomic surveillance, MDR *Klebsiella pneumoniae *surveillance, drug-resistant *Mycobacterium tuberculosis* surveillance, and data management. The methodological approaches, expected outcomes, capacity-building plans, data-sharing intentions and policy-engagement activities are sufficiently explained for an Open Letter.

 Overall, the article is scientifically sound, accessible, adequately referenced, and suitable for indexing.

Does the article adequately reference differing views and opinions?

Yes

Are all factual statements correct, and are statements and arguments made adequately supported by citations?

Yes

Is the Open Letter written in accessible language?

Yes

Where applicable, are recommendations and next steps explained clearly for others to follow?

Yes

Is the rationale for the Open Letter provided in sufficient detail?

Yes

Reviewer Expertise:

Pathogen genomics

I confirm that I have read this submission and believe that I have an appropriate level of expertise to confirm that it is of an acceptable scientific standard.

# Reviewer report: Reviewer response for version 1

**DOI:** 10.21956/wellcomeopenres.28217.r146799

**Published:** 2026-02-28

**Authors:** Marina Igorova Reyne

**Affiliations:** 1 Queen’s University Belfast, Belfast, UK

Summary: The open letter describes the formation of a new consortium involving several organizations across Africa and Asia, aimed at strengthening genomic surveillance for four high‑burden pathogens. Its goal is to enhance knowledge and monitoring of disease outbreaks and to integrate genomic data into management practices and policy development, enabling more informed and effective decision‑making.

 The manuscript would benefit from improvements to its structure to make it easier to follow. There is also considerable repetition throughout the manuscript, and it would benefit from being more concise. Clearer subheadings are needed, beginning with an Introduction that explains why the consortium is needed, defines the problem, and outlines the overall goals. This should be followed by distinct sections describing each work package with more details. In its current form, the manuscript feels somewhat limited in its discussion of capacity building and the anticipated impact on policy decisions. The section stating that the consortium will “engage regularly with national and subnational policymakers, fostering stronger collaboration and sharing project outcomes” would benefit from elaboration and could be expanded into a standalone section.

 Abstract: The pathogens discussed in the manuscript are chikungunya virus and dengue virus, which cause the diseases chikungunya and dengue, respectively. These names should be corrected and used consistently throughout the manuscript.

 Introduction: The abstract indicates that one focus of the study is on pathogens influenced by climate change, but this is not clearly reflected in the Introduction. Additional detail is needed on how climate change affects these pathogens, for example through expansion of mosquito vector distributions and increasing environmental suitability for flavivirus transmission. This context would strengthen the link between climate drivers and pathogen dynamics. When presenting each pathogen, the authors should provide a brief description of the disease it causes and separate the discussion into individual paragraphs for clarity. Information on the current availability of genomic data, as well as existing surveillance practices and policies relevant to each pathogen, should also be included. In addition, Klebsiella and tuberculosis should be separated into distinct paragraphs, as they represent different pathogen groups and public health challenges. The manuscript should maintain consistency in the use of terminology, choosing either abbreviations or full pathogen names and applying them uniformly throughout.

 Methodology: The section requires clearer organization to improve readability. It would benefit from being divided into work packages, with each one outlining the overall goal, methodological approach, and expected outcomes. For each pathogen, the manuscript should specify the sample types to be collected, how they will be processed in the laboratory, the sequencing methods to be used, the bioinformatics pipeline, and the planned downstream analyses. Tables should be numbered based on the order they are cited in the text. Expected outcomes should be presented in subsections dedicated to each pathogen. The manuscript currently refers to “Real-Time (RT)-quantitative PC,” but in this context RT should stand for Reverse Transcription. The approach for viral sequencing also needs clarification. Viral whole‑genome sequencing can be challenging, so the authors should specify which enrichment protocol or strategy will be used. For Klebsiella, it is unclear how whole‑genome sequences will be obtained, whether pure culture followed by shotgun sequencing will be used, and whether both short‑read and long‑read technologies will be combined for assembly. For the bacterial pathogens in general, the manuscript should clarify whether they will be cultured, tested for resistance, and then sequenced from pure isolates. The section on building sustainable local sequencing capacity should also be expanded to provide more detail.

 In Figure 1, WP1 appears to be missing, and captions for all figures require additional information.

Does the article adequately reference differing views and opinions?

Yes

Are all factual statements correct, and are statements and arguments made adequately supported by citations?

Yes

Is the Open Letter written in accessible language?

Partly

Where applicable, are recommendations and next steps explained clearly for others to follow?

Not applicable

Is the rationale for the Open Letter provided in sufficient detail?

Partly

Reviewer Expertise:

pathogen genomics, molecular biology, epidemiological modelling, disease ecology

I confirm that I have read this submission and believe that I have an appropriate level of expertise to confirm that it is of an acceptable scientific standard, however I have significant reservations, as outlined above.

# Response

**Published:** 2026-05-11

**Authors:** Patience Kiyuka

**Affiliations:** Bioscience, Kenya Medical Research Institute, Kilifi, Kenya

We thank the reviewer for the insightful comments, below is our response to the issues raised;

***The manuscript would benefit from improvements to its structure to make it easier to follow. There is also considerable repetition throughout the manuscript, and it would benefit from being more concise. Clearer subheadings are needed, beginning with an Introduction that explains why the consortium is needed, defines the problem, and outlines the overall goals. This should be followed by distinct sections describing each work package with more details.***

 We have reorganized the manuscript into distinct sections with clear subheadings. In the revised version of the manuscript there is the introduction, methodology which has all the work packages described followed by the opportunities, challenges and future directions and communication/engagement and dissemination plans.******

***In its current form, the manuscript feels somewhat limited in its discussion of capacity building and the anticipated impact on policy decisions. The section stating that the consortium will “engage regularly with national and subnational policymakers, fostering stronger collaboration and sharing project outcomes” would benefit from elaboration and could be expanded into a standalone section.***  

 We have added the following text under communication/engagement and dissemination plans; The overall aim is to strengthen national public health institutions by providing enhanced genomic surveillance data on priority pathogens and improving outbreak response mechanisms. To achieve this, we will engage regularly with national and subnational policymakers to foster stronger collaboration. At project initiation we will hold engagement meetings with subnational and national level policy makers to introduce the project objectives, planned activities, expected outputs and potential policy implications. These initial project engagement meetings will also serve to establish communication channels between the researchers and the policy makers. 

 Throughout the project, we will regularly share the data and results obtained.  Through WP 5, we will develop data dashboards based on the genomic surveillance and epidemiological data generated across the AAPs/ CIDRI-A. The dashboard will be made freely available to policy makers and the NPHL to support outbreak preparedness and response planning in addition to evidence-based decision making. The dashboard and the associated data tools will be handed over to the NPHL for project sustainability. Final project results generated from the consortium will also be translated into policy brief tailored to national and sub-national decision-makers. The policy briefs will be disseminated through structured policy engagement meetings, stakeholder workshops and to the relevant MoHs relevant technical working groups (TWGs). These engagements will provide a forum to discuss policy implications, foreseeable implementation challenges and recommendations for strengthening the regions outbreak response strategies.

**Abstract: *The pathogens discussed in the manuscript are chikungunya virus and dengue virus, which cause the diseases chikungunya and dengue, respectively. These names should be corrected and used consistently throughout the manuscript.***

 We have made this correction.  

**Introduction: *The abstract indicates that one focus of the study is on pathogens influenced by climate change, but this is not clearly reflected in the Introduction. Additional detail is needed on how climate change affects these pathogens, for example through expansion of mosquito vector distributions and increasing environmental suitability for flavivirus transmission. This context would strengthen the link between climate drivers and pathogen dynamics. ***  

 We thank the reviewer for this comment. We have added the following statements to the relevant sections;   Arbovirus distribution is predicted to be strongly affected by climate change. The distribution of the *Aedes *mosquito vectors responsible for transmission of dengue and chikungunya viruses have been extensively studied, investigating the effects of temperature  (Mordecai et al. 2017; Huber et al. 2018) and humidity  (Hales et al. 2002) on habitat suitability and suggesting a climate-driven expansion into new ranges (Campbell et al. 2015), and a corresponding increased population at risk from mosquito-borne disease (Messina et al. 2019).  We will aim to integrate with existing studies looking at the impact of climate change on our chosen pathogens. For dengue, the existing Dengue Advanced Readiness Tools consortium (DART) https://www.dartdengue.org, a separately funded project which is also lead by OUCRU-Vietnam, are integrating climatic factors into dengue outbreak forecasting, and we will work with them to expand their tools to sites in this consortium and to integrate genetic factors into their model.   For TB, climate change acts primarily through indirect pathways. It has been shown that climate change has potential to worsen the TB epidemic by among others contributing to population displacement which often leads to overcrowding living conditions that accelerates TB transmission (Goscé et al. 2025). Healthcare disruption can cause TB treatment interruption, leading to longer infectious period and ultimately increased risk of development of drug resistance (Saunders et al. 2026). Moreover climate-related and socioeconomic disruptions contribute to food insecurity and malnutrition, which weakens the immune system and increasing the risk of TB infection and transmission (Shadi et al. 2025).        

***When presenting each pathogen, the authors should provide a brief description of the disease it causes and separate the discussion into individual paragraphs for clarity. Information on the current availability of genomic data, as well as existing surveillance practices and policies relevant to each pathogen, should also be included. ***  

 We have added the following to capture current availability of genomic data, as well as existing surveillance practices and policies relevant to each pathogen.   Genomic data from dengue is available for many of the countries in the CAGED consortium, however this is not evenly distributed by country. We assessed the data used by the Nextstrain real-time tracking of viral evolution (Hadfield et al. 2018) to determine the availability of public sequence data for dengue and Chikungunya. While Thailand and Vietnam have over 2000 available dengue genomes deposited in public databases, the majority were sampled before 2020. For Laos, Nepal, and Kenya, there are fewer than 150 genomes per country, and for Indonesia there are 555 genomes. For Chikungunya, there are over 150 complete genomes for Thailand, but fewer than 50 for all other countries in this consortium, and no genomes for Vietnam or Nepal. Although some countries have existing surveillance policies for the pathogens in this consortium, others don’t. Thailand has a national disease surveillance program which includes case reporting for Chikungunya and dengue (Thawillarp 2025) viruses and XDR TB. Laos has a national surveillance program for dengue which only includes clinical case reporting.   Existing global surveillance platform for *Klebsiella pneumoniae* (KlebNET-GSP) was established in 2018, currently hosting 110,891 Kpn genomes with associated metadata (https://next.pathogen.watch/genomes/573-klebsiella-pneumoniae, assessed on 16th March 2026). This global platform also incorporates a set of standardized bioinformatic pipelines and genomic reporting frameworks, which greatly facilitate data harmonization and the interpretation of AMR genotypes. It further supports global efforts to develop a genomic risk framework, assess K and O antigen distributions and track the transmission of high-risk lineages associated with neonatal sepsis. However, national policies on Kpn genomic surveillance remain largely lacking across countries within the AAPs. For TB, large WGS datasets are now widely available, though no single platform tracks global totals. Key collections include the CRyPTIC dataset of 12,289 sequenced isolates (The CRyPTIC Consortium 2022), the Afro‑TB dataset with 17,641 strains (13,753 curated) from 26 African countries (Laamarti et al. 2023), and the NIAID TB Portals resource housing genomic‑linked data from over 28,000 TB cases https://www.niaid.nih.gov/research/tb-portals. Additional raw reads are deposited across the NCBI SRA https://sra-explorer.info and ENA https://www.ebi.ac.uk/ena/browser/home, the primary global repositories, although neither provides organism‑specific counts. Tools like Pathogenwatch https://next.pathogen.watch/en, CRyPTIC, and the TB‑Resources index https://github.com/a-r-j/TB-Resources help researchers identify and navigate these distributed. Despite the large volume of data, global coverage is uneven: most publicly available genomes derive from high‑income settings and dominant Eurasian lineages, while many regions - including much of Africa and parts of Asia and the Pacific - remain under sampled. All countries hosting AAP‑affiliated sites maintain National TB Programmes, but surveillance systems differ substantially in diagnostic reach and laboratory capacity. WHO regional analyses highlight persistent gaps in case detection and incomplete linkage of diagnostic data to national reporting systems in several high‑burden settings https://www.who.int/publications/i/item/9789240116924. Laboratory assessments similarly report limited access to culture, Drug-Susceptibility Testing (DST), and advanced molecular diagnostics across many countries (Iragena et al. 2025), despite wide uptake of Xpert MTB/RIF as the primary test for rifampicin resistance (Kohli et al. 2021). As a result, surveillance in many AAP‑region settings remains heavily Xpert‑based, providing drug‑resistance screening but lacking the resolution required for genomic epidemiology or monitoring emerging resistance.

***The manuscript should maintain consistency in the use of terminology, choosing either abbreviations or full pathogen names and applying them uniformly throughout.***

 Thank you for this comment. We have revised to ensure consistency.  

**Methodology *The section requires clearer organization to improve readability. It would benefit from being divided into work packages, with each one outlining the overall goal, methodological approach, and expected outcomes.***

 Thank you for this comment. We have restructured all pathogen work packages to include three sections: overall goal, methodological approach, and expected outcome. For the project management work package, we renamed "methodological approach" to "management and governance of the consortium." This wording reflects the content more accurately and makes the section easier for readers to navigate, as a methodological approach is more appropriate for the pathogen-specific work packages.  

***For each pathogen, the manuscript should specify the sample types to be collected, how they will be processed in the laboratory, the sequencing methods to be used, the bioinformatics pipeline, and the planned downstream analyses.***

 We have added the following text to provide clarity on the laboratory processing, sequencing and the bioinformatics pipeline that will be used. *CHIK and DENV work package* We will utilize retrospective and prospective confirmed (by Reverse Transcription (RT)-quantitative PCR CHIKV blood samples. Additional febrile surveillance retrospective and prospective samples, serum or plasma, will be screened by RT-qPCR for CHIKV and DENV. Sequencing and bioinformatics pipeline methodologies will be standardized across the sites. WGS of CHIKV and DENV will be done using amplicon-based approach using the Illumina or ONT platforms, according to the technique available on each site. Whole genomes will be first amplified by overlapping RT-PCR amplifying 4 to 8 long fragments in separated RT-PCRs, or small fragments in two pools of RT-PCRs, according to the procedures in place on each site. The RT-PCR products will then be pooled and cleaned, and the library will be built using either Rapid Barcoding or Native Barcoding ONT kits, or Illumina library preparation kits according to the technique available at each site. The read analysis will be performed using established pipelines such as the artic network amplicon pipeline https://artic.network/resources/amplicon-nf  which processes both Illumina and ONT data. While sites will not harmonise sequencing protocols, owing to different platform availability, we will standardize the data QC and metrics to ensure we use high quality sequences for the downstream analysis, and an internal EQA process will allow comparison of sequences between sites. *Kpn work package* We will use a single whole genome sequencing (WGS) standard operating procedure (SOP) and bioinformatics pipeline. All Kpn isolates identified from blood and CSF cultures will undergo routine antimicrobial susceptibility testing to support clinical care, and will subsequently be cultured for DNA extraction, using either the Wizard® Genomic DNA Purification Kit (Promega, USA) or the DNeasy Blood & Tissue Kit (Qiagen, Germany), depending on local availability. DNA libraries will be prepared using the Nextera XT DNA Library Preparation Kit or the NEBNext Ultra II FS DNA Library Prep Kit for Illumina. WGS will be done on a locally established Illumina platform (Miseq, Hiseq or Novaseq), with the OTN platform used for selected samples.   We will infer transmission of high-risk lineages using phylodynamic modelling and SNP analyses. SNP calling will be performed using the Snippy pipeline ( https://github.com/tseemann/snippy). Maximum likelihood phylogenies will be inferred from the core SNP alignment, using IQ-TREE v2.2.0.3 with best-fit substitution models and 1,000 ultrafast bootstraps and these phylogenetic trees will serve as the basic for time-scaled phylodynamic analysis using BEAST X (Didelot et al. 2018). *Mtb work package * Sputum will be collected from patients with RR-TB confirmed by Xpert MTB/RIF. Isolates will be cultured to purity in category 3 facilities and DNA extracted from pure isolates prior to Illumina/ONT sequencing. Consensus genomes and variant calling will use MTBseq (Kohl et al. 2018) or GPAS. Resistance mutations relevant to fluoroquinolones, bedaquiline, pretomanid, and linezolid will be identified using the WHO mutation catalogue, with phenotypic DST performed on isolates with mutations of uncertain significance.    

***Tables should be numbered based on the order they are cited in the text.***

 We have made this correction to the revised version of the manuscript.  

***Expected outcomes should be presented in subsections dedicated to each pathogen.***

 We have aligned expected outcomes to each pathogen work package in the revised manuscript.  

***The manuscript currently refers to “Real-Time (RT)-quantitative PC,” but in this context RT should stand for Reverse Transcription.***  

 We have made this correction to the revised version of the manuscript to read as reverse transcription.  

***The approach for viral sequencing also needs clarification. Viral whole‑genome sequencing can be challenging, so the authors should specify which enrichment protocol or strategy will be used.***

 We have made clarifications that WGS of CHIKV and DENV will be done using amplicon-based approach using the Illumina or ONT platforms according to the technique available on each site. Whole genomes will be first amplified by overlapping RT-PCR amplifying 4 to 8 long fragments in separated RT-PCRs, or small fragments in two pools of RT-PCRs, according to the procedures in place on each site. The RT-PCR products will then be pooled and cleaned, and the library will be built using either Rapid Barcoding or Native Barcoding ONT kits, or Illumina library preparation kits according to the technique available at each site. The read analysis will be performed using established pipelines such as the artic network amplicon pipeline https://artic.network/resources/amplicon-nf, which processes both Illumina and ONT data. While sites will not harmonise sequencing protocols, owing to different platform availability, we will standardize the data QC and metrics to ensure we use high quality sequences for the downstream analysis, and an internal EQA process will allow comparison of sequences between sites.    

***For Klebsiella, it is unclear how whole‑genome sequences will be obtained, whether pure culture followed by shotgun sequencing will be used, and whether both short‑read and long‑read technologies will be combined for assembly.***

 We have clarified in the text that we will use whole genome sequencing and that Kpn isolated obtained from blood or CSF will undergo culture before sequencing. We will not use shot-gun sequencing rather it will be whole genome sequencing from pure cultures. Except for South Africa, most sites have capacity for shot-read Illumina sequencing    

***For the bacterial pathogens in general, the manuscript should clarify whether they will be cultured, tested for resistance, and then sequenced from pure isolates.***

 We have clarified that sputum will be collected from patients with RR-TB confirmed by Xpert MTB/RIF. Isolates will be cultured to purity in category 3 laboratory facilities and DNA extracted from pure isolates prior to Illumina/ONT sequencing. *Kpn *isolates identification from blood and CSF cultures will undergo the routine antimicrobial susceptibility testing to support clinical care and will subsequently be cultured for DNA extraction then sequencing performed.  

***The section on building sustainable local sequencing capacity should also be expanded to provide more detail.***  

 We thank the reviewer for this comment. We have added the following information to provide more clarity on how we will build sustainable local sequencing capacity under the section on opportunities, challenges and future direction.   For the *Kpn*, we will leverage this cross-AAP platform to support national AMR reference laboratories in tracking the spread of carbapenem-resistant Kpn lineages across hospital surveillance systems (e.g in Vietnam) or to assist collaborative hospitals in confirming and responding to nosocomial Kpn outbreaks (e.g in Kenya). To build sustainable local sequencing capacity, for Mtb, CIDRI-A and OUCRU will transfer standardized protocols and provide hands-on training to AAPs that do not currently conduct these assays, including MLW and KWTRP, with SOPs shared across all sites. MORU and OUCRU (DENV) and KWTRP (CHIKV), based in their existing expertise, will lead the standardization of sequencing and bioinformatic pipeline methodologies across AAPs. SOPs will be shared and training provided, where needed, to support the deployment of the tools in each AAP.  

***In Figure 1, WP1 appears to be missing, and captions for all figures require additional information.*** We have included WP 1 in figure 1 and improved the captions across all the figures    

**References** Campbell, Lindsay P., Caylor Luther, David Moo-Llanes, Janine M. Ramsey, Rogelio Danis-Lozano, and A. Townsend Peterson. 2015. “Climate Change Influences on Global Distributions of Dengue and Chikungunya Virus Vectors.” *Philosophical Transactions of the Royal Society of London. Series B, Biological Sciences* (England) 370 (1665). https://doi.org/10.1098/rstb.2014.0135. Didelot, Xavier, Nicholas J. Croucher, Stephen D. Bentley, Simon R. Harris, and Daniel J. Wilson. 2018. “Bayesian Inference of Ancestral Dates on Bacterial Phylogenetic Trees.” *Nucleic Acids Research* 46 (22): e134. https://doi.org/10.1093/nar/gky783. Goscé, Lara, Julia M. Pescarini, Rein M. G. J. Houben, and Christopher Finn McQuaid. 2025. “Tuberculosis and the Climate Crisis in Latin America: A Predicament of Poverty, Migration and Displacement.” *BMJ Global Health* 10 (4): e018674. https://doi.org/10.1136/bmjgh-2024-018674. Hadfield, James, Colin Megill, Sidney M. Bell, et al. 2018. “Nextstrain: Real-Time Tracking of Pathogen Evolution.” *Bioinformatics* 34 (23): 4121–23. https://doi.org/10.1093/bioinformatics/bty407. Hales, Simon, Neil de Wet, John Maindonald, and Alistair Woodward. 2002. “Potential Effect of Population and Climate Changes on Global Distribution of Dengue Fever: An Empirical Model.” *Lancet (London, England)* (England) 360 (9336): 830–34. https://doi.org/10.1016/S0140-6736(02)09964-6. Huber, John H., Marissa L. Childs, Jamie M. Caldwell, and Erin A. Mordecai. 2018. “Seasonal Temperature Variation Influences Climate Suitability for Dengue, Chikungunya, and Zika Transmission.” *PLoS Neglected Tropical Diseases* 12 (5): e0006451. https://doi.org/10.1371/journal.pntd.0006451. Iragena, Jean de Dieu, Achilles Katamba, Dissou Affolabi, Moses Joloba, and Willy Ssengooba. 2025. “Tuberculosis Laboratory Capacity Building in the WHO African Region: The Past, the Present and the Future: A Viewpoint.” *PLOS Global Public Health* 5 (11): e0004979. https://doi.org/10.1371/journal.pgph.0004979. Kohl, Thomas Andreas, Christian Utpatel, Viola Schleusener, et al. 2018. “MTBseq: A Comprehensive Pipeline for Whole Genome Sequence Analysis of Mycobacterium Tuberculosis Complex Isolates.” *PeerJ* 6: e5895. https://doi.org/10.7717/peerj.5895. Kohli, Mikashmi, Ian Schiller, Nandini Dendukuri, et al. 2021. “Xpert MTB/RIF Ultra and Xpert MTB/RIF Assays for Extrapulmonary Tuberculosis and Rifampicin Resistance in Adults.” *The Cochrane Database of Systematic Reviews* 1 (1): CD012768. https://doi.org/10.1002/14651858.CD012768.pub3. Laamarti, Meriem, Yasmine El Fathi Lalaoui, Rachid Elfermi, Rachid Daoud, and Achraf El Allali. 2023. “Afro-TB Dataset as a Large Scale Genomic Data of Mycobacterium Tuberuclosis in Africa.” *Scientific Data* 10 (1): 212. https://doi.org/10.1038/s41597-023-02112-3. Messina, Jane P., Oliver J. Brady, Nick Golding, et al. 2019. “The Current and Future Global Distribution and Population at Risk of Dengue.” *Nature Microbiology* 4 (9): 1508–15. https://doi.org/10.1038/s41564-019-0476-8. Mordecai, Erin A., Jeremy M. Cohen, Michelle V. Evans, et al. 2017. “Detecting the Impact of Temperature on Transmission of Zika, Dengue, and Chikungunya Using Mechanistic Models.” *PLoS Neglected Tropical Diseases* (United States) 11 (4): e0005568. https://doi.org/10.1371/journal.pntd.0005568. Saunders, Matthew J., Delia Boccia, Palwasha Y. Khan, et al. 2026. “Climate Change and Tuberculosis: An Analytical Framework.” *The Lancet Respiratory Medicine* 14 (3): 267–80. https://doi.org/10.1016/S2213-2600(25)00329-7. Shadi, Yahya, Esmaeil Khedmati Morasae, Salman Khazaei, et al. 2025. “Understanding the Mechanisms of Climate Change Impact on Tuberculosis: A Complex Systems Approach.” *BMC Public Health* 25 (1): 3382. https://doi.org/10.1186/s12889-025-24709-6. Thawillarp, Supharerk. 2025. “Comparison of Data and Performance Indicators: Before and After the Transition of Thailand’s National Disease Surveillance System, 2023–2024.” *Outbreak, Surveillance, Investigation & Response (OSIR) Journal* 18 (2): 70–77. https://doi.org/10.59096/osir.v18i2.272949. The CRyPTIC Consortium. 2022. “A Data Compendium Associating the Genomes of 12,289 Mycobacterium Tuberculosis Isolates with Quantitative Resistance Phenotypes to 13 Antibiotics.” *PLoS Biology* 20 (8): e3001721. https://doi.org/10.1371/journal.pbio.3001721.

# Response

**Published:** 2026-05-14

**Authors:** Patience Kiyuka

**Affiliations:** Bioscience, Kenya Medical Research Institute, Kilifi, Kenya

***The manuscript would benefit from improvements to its structure to make it easier to follow. There is also considerable repetition throughout the manuscript, and it would benefit from being more concise. Clearer subheadings are needed, beginning with an Introduction that explains why the consortium is needed, defines the problem, and outlines the overall goals. This should be followed by distinct sections describing each work package with more details.***

 We have reorganized the manuscript into distinct sections with clear subheadings. In the revised version of the manuscript there is the introduction, methodology which has all the work packages described followed by the opportunities, challenges and future directions and communication/engagement and dissemination plans.******

***In its current form, the manuscript feels somewhat limited in its discussion of capacity building and the anticipated impact on policy decisions. The section stating that the consortium will “engage regularly with national and subnational policymakers, fostering stronger collaboration and sharing project outcomes” would benefit from elaboration and could be expanded into a standalone section.***  

 We have added the following text under communication/engagement and dissemination plans; The overall aim is to strengthen national public health institutions by providing enhanced genomic surveillance data on priority pathogens and improving outbreak response mechanisms. To achieve this, we will engage regularly with national and subnational policymakers to foster stronger collaboration. At project initiation we will hold engagement meetings with subnational and national level policy makers to introduce the project objectives, planned activities, expected outputs and potential policy implications. These initial project engagement meetings will also serve to establish communication channels between the researchers and the policy makers.  Throughout the project, we will regularly share the data and results obtained.  Through WP 5, we will develop data dashboards based on the genomic surveillance and epidemiological data generated across the AAPs/ CIDRI-A. The dashboard will be made freely available to policy makers and the NPHL to support outbreak preparedness and response planning in addition to evidence-based decision making. The dashboard and the associated data tools will be handed over to the NPHL for project sustainability. Final project results generated from the consortium will also be translated into policy brief tailored to national and sub-national decision-makers. The policy briefs will be disseminated through structured policy engagement meetings, stakeholder workshops and to the relevant MoHs relevant technical working groups (TWGs). These engagements will provide a forum to discuss policy implications, foreseeable implementation challenges and recommendations for strengthening the regions outbreak response strategies.

**Abstract: *The pathogens discussed in the manuscript are chikungunya virus and dengue virus, which cause the diseases chikungunya and dengue, respectively. These names should be corrected and used consistently throughout the manuscript.***

 We have made this correction. **Introduction: *The abstract indicates that one focus of the study is on pathogens influenced by climate change, but this is not clearly reflected in the Introduction. Additional detail is needed on how climate change affects these pathogens, for example through expansion of mosquito vector distributions and increasing environmental suitability for flavivirus transmission. This context would strengthen the link between climate drivers and pathogen dynamics. ***  

 We thank the reviewer for this comment. We have added the following statements to the relevant sections;   Arbovirus distribution is predicted to be strongly affected by climate change. The distribution of the *Aedes *mosquito vectors responsible for transmission of dengue and chikungunya viruses have been extensively studied, investigating the effects of temperature  (Mordecai et al. 2017; Huber et al. 2018) and humidity  (Hales et al. 2002) on habitat suitability and suggesting a climate-driven expansion into new ranges (Campbell et al. 2015), and a corresponding increased population at risk from mosquito-borne disease (Messina et al. 2019).  We will aim to integrate with existing studies looking at the impact of climate change on our chosen pathogens. For dengue, the existing Dengue Advanced Readiness Tools consortium (DART) https://www.dartdengue.org, a separately funded project which is also lead by OUCRU-Vietnam, are integrating climatic factors into dengue outbreak forecasting, and we will work with them to expand their tools to sites in this consortium and to integrate genetic factors into their model.   For TB, climate change acts primarily through indirect pathways. It has been shown that climate change has potential to worsen the TB epidemic by among others contributing to population displacement which often leads to overcrowding living conditions that accelerates TB transmission (Goscé et al. 2025). Healthcare disruption can cause TB treatment interruption, leading to longer infectious period and ultimately increased risk of development of drug resistance (Saunders et al. 2026). Moreover climate-related and socioeconomic disruptions contribute to food insecurity and malnutrition, which weakens the immune system and increasing the risk of TB infection and transmission (Shadi et al. 2025).        

***When presenting each pathogen, the authors should provide a brief description of the disease it causes and separate the discussion into individual paragraphs for clarity. Information on the current availability of genomic data, as well as existing surveillance practices and policies relevant to each pathogen, should also be included. ***  

 We have added the following to capture current availability of genomic data, as well as existing surveillance practices and policies relevant to each pathogen.   Genomic data from dengue is available for many of the countries in the CAGED consortium, however this is not evenly distributed by country. We assessed the data used by the Nextstrain real-time tracking of viral evolution (Hadfield et al. 2018) to determine the availability of public sequence data for dengue and Chikungunya. While Thailand and Vietnam have over 2000 available dengue genomes deposited in public databases, the majority were sampled before 2020. For Laos, Nepal, and Kenya, there are fewer than 150 genomes per country, and for Indonesia there are 555 genomes. For Chikungunya, there are over 150 complete genomes for Thailand, but fewer than 50 for all other countries in this consortium, and no genomes for Vietnam or Nepal. Although some countries have existing surveillance policies for the pathogens in this consortium, others don’t. Thailand has a national disease surveillance program which includes case reporting for Chikungunya and dengue (Thawillarp 2025) viruses and XDR TB. Laos has a national surveillance program for dengue which only includes clinical case reporting.   Existing global surveillance platform for *Klebsiella pneumoniae* (KlebNET-GSP) was established in 2018, currently hosting 110,891 Kpn genomes with associated metadata (https://next.pathogen.watch/genomes/573-klebsiella-pneumoniae, assessed on 16th March 2026). This global platform also incorporates a set of standardized bioinformatic pipelines and genomic reporting frameworks, which greatly facilitate data harmonization and the interpretation of AMR genotypes. It further supports global efforts to develop a genomic risk framework, assess K and O antigen distributions and track the transmission of high-risk lineages associated with neonatal sepsis. However, national policies on Kpn genomic surveillance remain largely lacking across countries within the AAPs. For TB, large WGS datasets are now widely available, though no single platform tracks global totals. Key collections include the CRyPTIC dataset of 12,289 sequenced isolates (The CRyPTIC Consortium 2022), the Afro‑TB dataset with 17,641 strains (13,753 curated) from 26 African countries (Laamarti et al. 2023), and the NIAID TB Portals resource housing genomic‑linked data from over 28,000 TB cases https://www.niaid.nih.gov/research/tb-portals. Additional raw reads are deposited across the NCBI SRA https://sra-explorer.info and ENA https://www.ebi.ac.uk/ena/browser/home, the primary global repositories, although neither provides organism‑specific counts. Tools like Pathogenwatch https://next.pathogen.watch/en, CRyPTIC, and the TB‑Resources index https://github.com/a-r-j/TB-Resources help researchers identify and navigate these distributed. Despite the large volume of data, global coverage is uneven: most publicly available genomes derive from high‑income settings and dominant Eurasian lineages, while many regions - including much of Africa and parts of Asia and the Pacific - remain under sampled. All countries hosting AAP‑affiliated sites maintain National TB Programmes, but surveillance systems differ substantially in diagnostic reach and laboratory capacity. WHO regional analyses highlight persistent gaps in case detection and incomplete linkage of diagnostic data to national reporting systems in several high‑burden settings https://www.who.int/publications/i/item/9789240116924. Laboratory assessments similarly report limited access to culture, Drug-Susceptibility Testing (DST), and advanced molecular diagnostics across many countries (Iragena et al. 2025), despite wide uptake of Xpert MTB/RIF as the primary test for rifampicin resistance (Kohli et al. 2021). As a result, surveillance in many AAP‑region settings remains heavily Xpert‑based, providing drug‑resistance screening but lacking the resolution required for genomic epidemiology or monitoring emerging resistance.

***The manuscript should maintain consistency in the use of terminology, choosing either abbreviations or full pathogen names and applying them uniformly throughout.***

 Thank you for this comment. We have revised to ensure consistency.  

**Methodology *The section requires clearer organization to improve readability. It would benefit from being divided into work packages, with each one outlining the overall goal, methodological approach, and expected outcomes.***

 Thank you for this comment. We have restructured all pathogen work packages to include three sections: overall goal, methodological approach, and expected outcome. For the project management work package, we renamed "methodological approach" to "management and governance of the consortium." This wording reflects the content more accurately and makes the section easier for readers to navigate, as a methodological approach is more appropriate for the pathogen-specific work packages.  

***For each pathogen, the manuscript should specify the sample types to be collected, how they will be processed in the laboratory, the sequencing methods to be used, the bioinformatics pipeline, and the planned downstream analyses.*** We have added the following text to provide clarity on the laboratory processing, sequencing and the bioinformatics pipeline that will be used. *CHIK and DENV work package* We will utilize retrospective and prospective confirmed (by Reverse Transcription (RT)-quantitative PCR CHIKV blood samples. Additional febrile surveillance retrospective and prospective samples, serum or plasma, will be screened by RT-qPCR for CHIKV and DENV. Sequencing and bioinformatics pipeline methodologies will be standardized across the sites. WGS of CHIKV and DENV will be done using amplicon-based approach using the Illumina or ONT platforms, according to the technique available on each site. Whole genomes will be first amplified by overlapping RT-PCR amplifying 4 to 8 long fragments in separated RT-PCRs, or small fragments in two pools of RT-PCRs, according to the procedures in place on each site. The RT-PCR products will then be pooled and cleaned, and the library will be built using either Rapid Barcoding or Native Barcoding ONT kits, or Illumina library preparation kits according to the technique available at each site. The read analysis will be performed using established pipelines such as the artic network amplicon pipeline https://artic.network/resources/amplicon-nf  which processes both Illumina and ONT data. While sites will not harmonise sequencing protocols, owing to different platform availability, we will standardize the data QC and metrics to ensure we use high quality sequences for the downstream analysis, and an internal EQA process will allow comparison of sequences between sites. *Kpn work package* We will use a single whole genome sequencing (WGS) standard operating procedure (SOP) and bioinformatics pipeline. All Kpn isolates identified from blood and CSF cultures will undergo routine antimicrobial susceptibility testing to support clinical care, and will subsequently be cultured for DNA extraction, using either the Wizard® Genomic DNA Purification Kit (Promega, USA) or the DNeasy Blood & Tissue Kit (Qiagen, Germany), depending on local availability. DNA libraries will be prepared using the Nextera XT DNA Library Preparation Kit or the NEBNext Ultra II FS DNA Library Prep Kit for Illumina. WGS will be done on a locally established Illumina platform (Miseq, Hiseq or Novaseq), with the OTN platform used for selected samples.   We will infer transmission of high-risk lineages using phylodynamic modelling and SNP analyses. SNP calling will be performed using the Snippy pipeline ( https://github.com/tseemann/snippy). Maximum likelihood phylogenies will be inferred from the core SNP alignment, using IQ-TREE v2.2.0.3 with best-fit substitution models and 1,000 ultrafast bootstraps and these phylogenetic trees will serve as the basic for time-scaled phylodynamic analysis using BEAST X (Didelot et al. 2018). *Mtb work package * Sputum will be collected from patients with RR-TB confirmed by Xpert MTB/RIF. Isolates will be cultured to purity in category 3 facilities and DNA extracted from pure isolates prior to Illumina/ONT sequencing. Consensus genomes and variant calling will use MTBseq (Kohl et al. 2018) or GPAS. Resistance mutations relevant to fluoroquinolones, bedaquiline, pretomanid, and linezolid will be identified using the WHO mutation catalogue, with phenotypic DST performed on isolates with mutations of uncertain significance.    

***Tables should be numbered based on the order they are cited in the text.***

 We have made this correction to the revised version of the manuscript.  

***Expected outcomes should be presented in subsections dedicated to each pathogen. *** We have aligned expected outcomes to each pathogen work package in the revised manuscript.  

***The manuscript currently refers to “Real-Time (RT)-quantitative PC,” but in this context RT should stand for Reverse Transcription.***  

 We have made this correction to the revised version of the manuscript to read as reverse transcription.  

***The approach for viral sequencing also needs clarification. Viral whole‑genome sequencing can be challenging, so the authors should specify which enrichment protocol or strategy will be used.***

 We have made clarifications that WGS of CHIKV and DENV will be done using amplicon-based approach using the Illumina or ONT platforms according to the technique available on each site. Whole genomes will be first amplified by overlapping RT-PCR amplifying 4 to 8 long fragments in separated RT-PCRs, or small fragments in two pools of RT-PCRs, according to the procedures in place on each site. The RT-PCR products will then be pooled and cleaned, and the library will be built using either Rapid Barcoding or Native Barcoding ONT kits, or Illumina library preparation kits according to the technique available at each site. The read analysis will be performed using established pipelines such as the artic network amplicon pipeline https://artic.network/resources/amplicon-nf, which processes both Illumina and ONT data. While sites will not harmonise sequencing protocols, owing to different platform availability, we will standardize the data QC and metrics to ensure we use high quality sequences for the downstream analysis, and an internal EQA process will allow comparison of sequences between sites.    

***For Klebsiella, it is unclear how whole‑genome sequences will be obtained, whether pure culture followed by shotgun sequencing will be used, and whether both short‑read and long‑read technologies will be combined for assembly.***

 We have clarified in the text that we will use whole genome sequencing and that Kpn isolated obtained from blood or CSF will undergo culture before sequencing. We will not use shot-gun sequencing rather it will be whole genome sequencing from pure cultures. Except for South Africa, most sites have capacity for shot-read Illumina sequencing    

***For the bacterial pathogens in general, the manuscript should clarify whether they will be cultured, tested for resistance, and then sequenced from pure isolates.***

 We have clarified that sputum will be collected from patients with RR-TB confirmed by Xpert MTB/RIF. Isolates will be cultured to purity in category 3 laboratory facilities and DNA extracted from pure isolates prior to Illumina/ONT sequencing. *Kpn *isolates identification from blood and CSF cultures will undergo the routine antimicrobial susceptibility testing to support clinical care and will subsequently be cultured for DNA extraction then sequencing performed.  

***The section on building sustainable local sequencing capacity should also be expanded to provide more detail.***  

 We thank the reviewer for this comment. We have added the following information to provide more clarity on how we will build sustainable local sequencing capacity under the section on opportunities, challenges and future direction.   For the *Kpn*, we will leverage this cross-AAP platform to support national AMR reference laboratories in tracking the spread of carbapenem-resistant Kpn lineages across hospital surveillance systems (e.g in Vietnam) or to assist collaborative hospitals in confirming and responding to nosocomial Kpn outbreaks (e.g in Kenya). To build sustainable local sequencing capacity, for Mtb, CIDRI-A and OUCRU will transfer standardized protocols and provide hands-on training to AAPs that do not currently conduct these assays, including MLW and KWTRP, with SOPs shared across all sites. MORU and OUCRU (DENV) and KWTRP (CHIKV), based in their existing expertise, will lead the standardization of sequencing and bioinformatic pipeline methodologies across AAPs. SOPs will be shared and training provided, where needed, to support the deployment of the tools in each AAP.  

***In Figure 1, WP1 appears to be missing, and captions for all figures require additional information.***

 We have included WP 1 in figure 1 and improved the captions across all the figures **References** Campbell, Lindsay P., Caylor Luther, David Moo-Llanes, Janine M. Ramsey, Rogelio Danis-Lozano, and A. Townsend Peterson. 2015. “Climate Change Influences on Global Distributions of Dengue and Chikungunya Virus Vectors.” *Philosophical Transactions of the Royal Society of London. Series B, Biological Sciences* (England) 370 (1665). https://doi.org/10.1098/rstb.2014.0135. Didelot, Xavier, Nicholas J. Croucher, Stephen D. Bentley, Simon R. Harris, and Daniel J. Wilson. 2018. “Bayesian Inference of Ancestral Dates on Bacterial Phylogenetic Trees.” *Nucleic Acids Research* 46 (22): e134. https://doi.org/10.1093/nar/gky783. Goscé, Lara, Julia M. Pescarini, Rein M. G. J. Houben, and Christopher Finn McQuaid. 2025. “Tuberculosis and the Climate Crisis in Latin America: A Predicament of Poverty, Migration and Displacement.” *BMJ Global Health* 10 (4): e018674. https://doi.org/10.1136/bmjgh-2024-018674. Hadfield, James, Colin Megill, Sidney M. Bell, et al. 2018. “Nextstrain: Real-Time Tracking of Pathogen Evolution.” *Bioinformatics* 34 (23): 4121–23. https://doi.org/10.1093/bioinformatics/bty407. Hales, Simon, Neil de Wet, John Maindonald, and Alistair Woodward. 2002. “Potential Effect of Population and Climate Changes on Global Distribution of Dengue Fever: An Empirical Model.” *Lancet (London, England)* (England) 360 (9336): 830–34. https://doi.org/10.1016/S0140-6736(02)09964-6. Huber, John H., Marissa L. Childs, Jamie M. Caldwell, and Erin A. Mordecai. 2018. “Seasonal Temperature Variation Influences Climate Suitability for Dengue, Chikungunya, and Zika Transmission.” *PLoS Neglected Tropical Diseases* 12 (5): e0006451. https://doi.org/10.1371/journal.pntd.0006451. Iragena, Jean de Dieu, Achilles Katamba, Dissou Affolabi, Moses Joloba, and Willy Ssengooba. 2025. “Tuberculosis Laboratory Capacity Building in the WHO African Region: The Past, the Present and the Future: A Viewpoint.” *PLOS Global Public Health* 5 (11): e0004979. https://doi.org/10.1371/journal.pgph.0004979. Kohl, Thomas Andreas, Christian Utpatel, Viola Schleusener, et al. 2018. “MTBseq: A Comprehensive Pipeline for Whole Genome Sequence Analysis of Mycobacterium Tuberculosis Complex Isolates.” *PeerJ* 6: e5895. https://doi.org/10.7717/peerj.5895. Kohli, Mikashmi, Ian Schiller, Nandini Dendukuri, et al. 2021. “Xpert MTB/RIF Ultra and Xpert MTB/RIF Assays for Extrapulmonary Tuberculosis and Rifampicin Resistance in Adults.” *The Cochrane Database of Systematic Reviews* 1 (1): CD012768. https://doi.org/10.1002/14651858.CD012768.pub3. Laamarti, Meriem, Yasmine El Fathi Lalaoui, Rachid Elfermi, Rachid Daoud, and Achraf El Allali. 2023. “Afro-TB Dataset as a Large Scale Genomic Data of Mycobacterium Tuberuclosis in Africa.” *Scientific Data* 10 (1): 212. https://doi.org/10.1038/s41597-023-02112-3. Messina, Jane P., Oliver J. Brady, Nick Golding, et al. 2019. “The Current and Future Global Distribution and Population at Risk of Dengue.” *Nature Microbiology* 4 (9): 1508–15. https://doi.org/10.1038/s41564-019-0476-8. Mordecai, Erin A., Jeremy M. Cohen, Michelle V. Evans, et al. 2017. “Detecting the Impact of Temperature on Transmission of Zika, Dengue, and Chikungunya Using Mechanistic Models.” *PLoS Neglected Tropical Diseases* (United States) 11 (4): e0005568. https://doi.org/10.1371/journal.pntd.0005568. Saunders, Matthew J., Delia Boccia, Palwasha Y. Khan, et al. 2026. “Climate Change and Tuberculosis: An Analytical Framework.” *The Lancet Respiratory Medicine* 14 (3): 267–80. https://doi.org/10.1016/S2213-2600(25)00329-7. Shadi, Yahya, Esmaeil Khedmati Morasae, Salman Khazaei, et al. 2025. “Understanding the Mechanisms of Climate Change Impact on Tuberculosis: A Complex Systems Approach.” *BMC Public Health* 25 (1): 3382. https://doi.org/10.1186/s12889-025-24709-6. Thawillarp, Supharerk. 2025. “Comparison of Data and Performance Indicators: Before and After the Transition of Thailand’s National Disease Surveillance System, 2023–2024.” *Outbreak, Surveillance, Investigation & Response (OSIR) Journal* 18 (2): 70–77. https://doi.org/10.59096/osir.v18i2.272949. The CRyPTIC Consortium. 2022. “A Data Compendium Associating the Genomes of 12,289 Mycobacterium Tuberculosis Isolates with Quantitative Resistance Phenotypes to 13 Antibiotics.” *PLoS Biology* 20 (8): e3001721. https://doi.org/10.1371/journal.pbio.3001721.

# Reviewer report: Reviewer response for version 1

**DOI:** 10.21956/wellcomeopenres.28217.r146802

**Published:** 2026-02-27

**Authors:** Rakesh K Mishra

**Affiliations:** 1 Tata Institute for Genetics and Society, Bengaluru, Karnataka, India

This manuscript details the establishment and operational framework of the Cross-Wellcome Africa Asian Programmes (AAPs) Acceleration of Genomics for Escalating Infectious Diseases (CAGED) Consortium. The consortium’s strength lies in its ability to leverage expertise across six major institutions in Africa and Southeast Asia. This geographic diversity allows for a unique "multi-site and multi-pathogen approach" that is essential for understanding the heterogeneous nature of infectious diseases in these regions. Furthermore, the focus on chikungunya, dengue, multidrug-resistant *Klebsiella pneumoniae*, and drug-resistant Mtb is highly relevant, particularly as these diseases are increasingly driven by climate change and pose significant public health threats.

 The manuscript clearly outlines the use of both Illumina and Oxford Nanopore Technology platforms. This dual-platform approach is a robust choice for handling the diverse genomic architectures of viruses (CHIKV/DENV) and bacteria (Kpn/Mtb). The reliance on established pipelines such as Kleborate for Kpn and MTBseq for Mtb ensures that the data analysis is standardized and scientifically rigorous.

 The manuscript emphasizes FAIR (Findable, Accessible, Interoperable, and Reusable) data principles. The plan to use the HL7 Fast Healthcare Interoperability Resources (FHIR) standard is a particularly forward-thinking approach to ensure data can be integrated with national health dashboards. A central goal is the transfer of capacity to National Public Health Laboratories and Ministries of Health. This ensures that the research doesn't remain academic but directly informs evidence-based policy making.

 Points to be considered:

 The consortium repeatedly states that it focuses on pathogens "driven by climate change". However, the provided methodology does not explicitly detail how environmental, meteorological, or climate data will be integrated with the genomic data . Without a specific analytical plan to correlate genomic shifts with climate variables, the "climate-driven" aspect remains a thematic justification rather than a core scientific inquiry.

 For the CHIKV and DENV work package, the plan to select a "minimum of 20 participant samples per group" for assessing neutralizing antibodies may lack sufficient statistical power . Given the high genetic diversity and the goal of assessing "immune escape" across multiple lineages in both Africa and Asia, such a small sample size might not be representative of the broader population immunity or the full spectrum of viral variants

 The manuscript justifies the project by noting the "limited use of genomics" for these pathogens compared to the 3 million SARS-CoV-2 genomes on GISAID . However, it does not fully address how the consortium will overcome the specific biological challenges—such as the low symptomatic-to-asymptomatic ratio in Dengue which makes representative genomic surveillance significantly more difficult.

 Overall, the manuscript presents a well-coordinated plan to enhance genomic surveillance in regions with historically limited resources compared to the global response to SARS-CoV-2.

Does the article adequately reference differing views and opinions?

Yes

Are all factual statements correct, and are statements and arguments made adequately supported by citations?

Yes

Is the Open Letter written in accessible language?

Yes

Where applicable, are recommendations and next steps explained clearly for others to follow?

Partly

Is the rationale for the Open Letter provided in sufficient detail?

Yes

Reviewer Expertise:

Environmenal surveillance, Genomics, epigenetics

I confirm that I have read this submission and believe that I have an appropriate level of expertise to confirm that it is of an acceptable scientific standard.

# Response

**Published:** 2026-05-11

**Authors:** Patience Kiyuka

**Affiliations:** Bioscience, Kenya Medical Research Institute, Kilifi, Kenya

We thank the reviewer for the thoughtful and sightful comments that have improved the rigor of our manuscript. Below is our responses to the comments:

***The consortium repeatedly states that it focuses on pathogens "driven by climate change". However, the provided methodology does not explicitly detail how environmental, meteorological, or climate data will be integrated with the genomic data . Without a specific analytical plan to correlate genomic shifts with climate variables, the "climate-driven" aspect remains a thematic justification rather than a core scientific inquiry.***  

 We sincerely thank the reviewer for pointing it out. We agree that not all the pathogens we are studying are directly driven by climate change and because we don’t have a dedicated methodological section where we provide details of how climatic variables will be incorporated into the genomic data except for DENV and CHIKV, we have changed the abstract to reflect that climate change is one of the factors driving transmission of some of these pathogens with the statement *“with climate change considered one of the several factors influencing the distribution, transmission or burden of some of these pathogens.”* We have also added the following text to the appropriate sections of the manuscript.   Arbovirus distribution is predicted to be strongly affected by climate change. The distribution of the *Aedes *mosquito vectors responsible for transmission of dengue and chikungunya viruses have been extensively studied, investigating the effects of temperature  (Mordecai et al. 2017; Huber et al. 2018) and humidity  (Hales et al. 2002) on habitat suitability and suggesting a climate-driven expansion into new ranges (Campbell et al. 2015), and a corresponding increased population at risk from mosquito-borne disease (Messina et al. 2019).       We will aim to integrate with existing studies looking at the impact of climate change on our chosen pathogens. For dengue, the existing Dengue Advanced Readiness Tools consortium (DART) https://www.dartdengue.org, a separately funded project which is also lead by OUCRU-Vietnam, are integrating climatic factors into dengue outbreak forecasting, and we will work with them to expand their tools to sites in this consortium and to integrate genetic factors into their model.   For TB, climate change acts primarily through indirect pathways. It has been shown that climate change has potential to worsen the TB epidemic by among others contributing to population displacement which often leads to overcrowding living conditions that accelerates TB transmission (Goscé et al. 2025). Healthcare disruption can cause TB treatment interruption, leading to longer infectious period and ultimately increased risk of development of drug resistance (Saunders et al. 2026). Moreover climate-related and socioeconomic disruptions contribute to food insecurity and malnutrition, which weakens the immune system and increasing the risk of TB infection and transmission (Shadi et al. 2025).  

***For the CHIKV and DENV work package, the plan to select a "minimum of 20 participant samples per group" for assessing neutralizing antibodies may lack sufficient statistical power. Given the high genetic diversity and the goal of assessing "immune escape" across multiple lineages in both Africa and Asia, such a small sample size might not be representative of the broader population immunity or the full spectrum of viral variants.***

   Thank you for the thoughtful comment regarding the proposed sample size of approximately 20 participants per group for assessing neutralizing antibodies against CHIKV and DENV. We carefully considered the concern about statistical power, representativeness of population immunity, and viral diversity across African and Asian lineages.  We have address this by adding the paragraph below   The selection of a minimum of 20 participant samples per group was based on a formal sample size determination conducted specifically for neutralization assays comparing responses between viral strains. A bootstrap-based power analysis was performed assuming neutralization titres (FRNT50) follow a normal distribution with high inter-sera variability (σ ≈ μ), which is typical for neutralization assays. The analysis further assumed that biologically meaningful differences correspond to approximately a three-fold difference in neutralization titres between strains. Using 1000 bootstrap iterations and a two-sided t-test with α = 0.05, the analysis demonstrated that approximately 20 sera samples are sufficient to detect such differences with statistical power ≥ 0.8.    Importantly, the purpose of this component is not to estimate population-level seroprevalence or overall immunity, but rather to experimentally compare neutralization capacity across viral lineages and assess potential immune escape at the virological and immunological level.  

***The manuscript justifies the project by noting the "limited use of genomics" for these pathogens compared to the 3 million SARS-CoV-2 genomes on GISAID. However, it does not fully address how the consortium will overcome the specific biological challenges—such as the low symptomatic-to-asymptomatic ratio in Dengue which makes representative genomic surveillance significantly more difficult.***

 We acknowledge the limitation of not including asymptomatic dengue cases; however, this study is based on existing surveillance of clinical cases in health facilities across the different sites and therefore does not include sequencing of asymptomatic cases. The focal area of the project is to expand the use of genomics in these pathogens, once it is routine it can be deployed on other samples sets as they become available from community surveys. We chose to start with all the pathogens through surveillance in health facilities where it can also support diagnostic detection where this is not available.
